# An astronomical question answering dataset for evaluating large language models

**DOI:** 10.1038/s41597-025-04613-9

**Published:** 2025-03-18

**Authors:** Jie Li, Fuyong Zhao, Panfeng Chen, Jiafu Xie, Xiangrui Zhang, Hui Li, Mei Chen, Yanhao Wang, Ming Zhu

**Affiliations:** 1 State Key Laboratory of Public Big Data, College of Computer Science and Technology, Guizhou University, Guiyang, 550025 China; 2 School of Data Science and Engineering, East China Normal University, Shanghai, 200062 China; 3 National Astronomical Observatories, Chinese Academy of Sciences, Beijing, 100101 China

**Keywords:** Information technology, Computer science

## Abstract

Large language models (LLMs) have recently demonstrated exceptional capabilities across a variety of linguistic tasks including question answering (QA). However, it remains challenging to assess their performance in astronomical QA due to the lack of comprehensive benchmark datasets. To bridge this gap, we construct Astro-QA, the first benchmark dataset specifically for QA in astronomy. The dataset contains a collection of 3,082 questions of six types in both English and Chinese, along with standard (reference) answers and related material. These questions encompass several core branches of astronomy, including *astrophysics*, *astrometry*, *celestial mechanics*, *history of astronomy*, and *astronomical techniques and methods*. Furthermore, we propose a new measure called *DGscore* that integrates different measures for objective and subjective questions and incorporates a weighting scheme based on type- and question-specific difficulty coefficients to accurately assess the QA performance of each LLM. We validate the Astro-QA dataset through extensive experimentation with 27 open-source and commercial LLMs. The results show that it can serve as a reliable benchmark dataset to evaluate the capacity of LLM in terms of instruction following, knowledge reasoning, and natural language generation in the astronomical domain, which can calibrate current progress and facilitate future research of astronomical LLMs.

## Background & Summary

In recent years, large language models (LLMs), such as GPT-4^1^, LLaMA^2^, and GLM^3^, have gained tremendous success, driving a paradigm shift to prompt-based generative artificial intelligence (GenAI) across a variety of tasks. Due to the remarkable understanding and reasoning capacities of LLMs, great effort has been devoted to applying them to linguistic tasks, especially question answering (QA), in specialized domains, such as biology^4^, medicine^5^, and law^6^. In this paper, we focus on applying LLMs to QA in astronomy. Particularly, several specialized language models in astronomy have been proposed, e.g., astroBERT^7^, AstroLLaMA^8,9^, AstroMLab^10,11^, and StarWhisper^12^. These models adopt pre-trained general-purpose LLMs like BERT^13^, LLaMA^2^, and GLM^3^ as foundation models and fine-tune them with scientific papers in astronomy. With excellent performance on discrimination and text generation tasks, they are deemed to have substantial knowledge of astronomy. Furthermore, Leung *et al*.^14^ pioneered an LLM-based framework for data-driven astronomy, demonstrating that a single LLM could perform both discriminative and generative tasks (e.g., distinguishing and generating stellar spectra), even without task-specific training or fine-tuning, which underscored its immense potential application in astronomy research. Ciucua *et al*.^15^ also investigated the ability of GPT-4 to interpret the literature in astronomy. Using contextual and adversarial prompts, they improved the efficiency of hypothesis generation in astronomy using GPT-4. Despite the considerable progress that has been achieved, these existing studies have not fully evaluated the QA performance of LLMs in astronomy.

With the rapid development of LLMs, a large number of benchmark datasets have been proposed to evaluate their performance in recent years. Wang *et al*.^16^ proposed the GLUE benchmark to evaluate the performance of natural language understanding (NLU) models. Srivastava *et al*.^17^ proposed BIG-Bench, a benchmark consisting of 204 tasks that are believed to be beyond the capabilities of current language models. AGIEval^18^ and GAOKAO^19^ used standardized exams, such as college entrance exams, law school admission tests, and lawyer qualification tests, to evaluate the performance of LLMs. Huang *et al*.^20^ proposed the C-Eval comprising 13,948 multiple choice questions spanning 52 subjects. These benchmarks do not involve any task in the astronomy domain. Furthermore, MMLU^21^, CMMLU^22^, and Xiezhi^23^ are also LLM benchmarks consisting of multiple choice questions, some of which (~170) are about astronomy. We note that CMMLU and Xiezhi are not fully available to the public. Zeng *et al*.^24^ proposed CG-Eval, a comprehensive benchmark for the text generation capacity of LLMs across multiple disciplines, where 100 terminology and 100 short-answer questions in the astronomy domain are included. However, as shown in Table 1, although some of these benchmarks contain questions about astronomy, they still suffer from several serious problems when used for the evaluation of LLMs for QA in astronomy. First, they rarely cover the terminology, concepts, and complex questions in the astronomy domain. In these benchmarks, astronomical questions are mostly about *common knowledge* that can be answered without specialization. Such questions cannot measure whether the LLM has actually acquired in-depth knowledge of astronomy. Second, their evaluations do not take into account the difficulty of the question and do not provide an integrated score to reflect the overall QA performance of an LLM for various types of questions. Third, they do not give a detailed explanation of the procedure for obtaining a given answer. As a result, users cannot understand how and why particular questions are correctly answered, whereas others are not.

**Table 1:** Comparison of Astro-QA with existing benchmarks. The abbreviations of six question types are: “TM” for *terminology*, “SQ” for *short-answer*, “SMC” for *single-select multiple choice*, “MMC” for *multi-select multiple choice*, “JU” for *judgment*, and “MT” for *matching*.

Name	Language	TM	SQ	SMC	MMC	JU	MT
GLUE^16^	en	0	0	0	0	0	0
BIG-Bench^17^	en	0	0	0	0	0	0
AGI-Eval^18^	zh	0	0	0	0	0	0
C-Eval^20^	zh	0	0	0	0	0	0
GAOKAO^19^	zh	0	0	0	0	0	0
MMLU^21^	en	0	0	173	0	0	0
CMMLU^22^	zh	0	0	170	0	0	0
Xiezhi^23^	zh	0	0	0	≪ 1%	0	0
CG-Eval^24^	zh	100	100	0	0	0	0
Astro-QA	en/zh	323	231	1699	157	317	355

The number of questions of each type only counts those related to the astronomy domain.

**Fig. 1:**
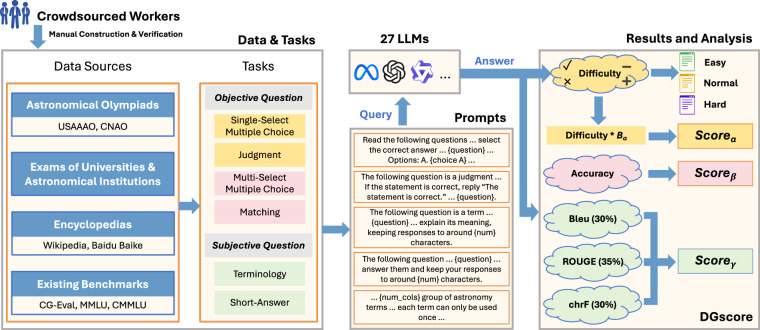
Overview of the Astro-QA benchmark dataset.

To address these problems, we propose Astro-QA, the first question answering dataset for LLMs specifically in astronomy. Fig. 1 presents an overall framework of the Astro-QA benchmark dataset. Astro-QA contains a collection of 3,082 questions from various sources. In addition to the questions obtained from established benchmarks^21,22,24^, we also collect public questions from the International Olympiad on Astronomy and Astrophysics (IOAA) competitions held in China and the United States, final exams of universities and institutions, as well as online encyclopedias such as Wikipedia and Baidu Baike. These questions encompass the five core subdomains of astronomy: *astrophysics*, *astrometry*, *celestial mechanics*, *history of astronomy*, and *astronomical techniques and methods*. Furthermore, Astro-QA features six different question types: *single-select multiple choice* (SMC), *multi-select multiple choice* (MMC), *judgment* (JU), *matching* (MT), *terminology* (TM) and *short-answer* (SQ). Notably, the matching questions in Astro-QA are created manually, as this type of astronomical question has not been included in any existing benchmark. In general, Astro-QA comprises a representative and diverse suite of questions in astronomy, allowing users to evaluate the QA performance of an LLM comprehensively. Then, according to pre-specified templates, Astro-QA translates each question into a prompt and feeds it to LLMs for answers. The answers are finally evaluated by *DGscore*, a novel measure of QA quality that integrates a wide spectrum of measures for different types of objective and subjective questions and incorporates a weighting scheme based on type- and question-specific difficulty coefficients. Along with the evaluation results, Astro-QA also presents an explanation of how the answer is reached, which is built manually by crowdsourced workers with solid astronomical knowledge. This can help users understand why an LLM successfully answers a specific question or not.

Finally, we conduct extensive experiments to validate the effectiveness of Astro-QA. Specifically, we employ a zero-shot approach for QA by querying 27 open-source and commercial LLMs using elaborate prompts. We rank all LLMs tested according to the DGscore and present more detailed results of their performance on questions of each type at each level of difficulty. The results show that LLMs have obtained common knowledge of astronomy even without specification, owing to their strong information extraction and generalization ability. However, when faced with difficult questions that require an in-depth understanding of astronomical terminology and concepts or utilize astronomical formulae for computation, all LLMs tested exhibit much inferior performance, indicating that their reasoning and mathematical capabilities in astronomy still have a lot of room for improvement. In summary, we hope that Astro-QA can calibrate the current progress and facilitate future research of astronomical LLMs.

## Methods

In this section, we provide a detailed description of the construction process of the Astro-QA dataset.

### Question Type

Our main motivation for proposing Astro-QA is to comprehensively assess the overall performance of LLMs for QA in astronomy. To achieve this goal, it contains the following six types of questions to evaluate the capacity of LLMs in different aspects.

- **Judgment (JU)**: This type of question asks an LLM to judge whether a statement is correct. It requires the LLM to distinguish factual knowledge in astronomy from non-factual knowledge. The LLM should understand the question and its corresponding astronomical concepts, theories, and facts and match them with each other. In addition, the LLM should also use its reasoning skills to detect possible errors and contradictions in the statement.
- **Single-Select Multiple-Choice (SMC)**: This type of question asks an LLM to pick the correct answer from four or five candidates. It requires the LLM to apply their knowledge of astronomy to analyze and select the most relevant answer among similar or related options. Some questions of this kind also evaluate the basic mathematical skill of the LLM since their answers can only be calculated by applying established formulae.
- **Multi-Select Multiple-Choice (MMC)**: This type of question is similar to SMC. However, more than one answer might be correct, and all correct answers should be chosen. In addition to the capacity for SMC, it also requires the LLM to understand the knowledge of astronomy in an associative manner because several different yet correlated concepts are considered in a single question. The LLM should cover all the concepts involved and analyze their correlation to identify all the correct answers.
- **Matching (MT)**: This type of question asks an LLM to build a one-to-one match between two sets of statements, where each statement in one set should be matched with its most relevant one in the other set. It requires the LLM to integrate multiple pieces of knowledge and accurately build their causal relationships.
- **Short-Answer (SQ)**: This type of question requires the LLM to write a paragraph to answer the question. The LLM needs, on the one hand, to understand the question itself and, on the other hand, to generate logical, organized, and concise text in response to the question.
- **Terminology (TM)**: This type of question asks the LLM to write a paragraph to describe a given concept. Since astronomical concepts are rarely used in daily life, it requires the LLM to specialize in the astronomy domain so as to memorize the terminology. Similarly to SQ, TM also requires that the generated text is logical, organized, and concise. In some cases, intuitive examples are also needed to explain the terminology.

### Data Sources

The questions in Astro-QA are collected from the following five sources: *(i)* existing benchmarks, *(ii)* astronomy Olympiad competitions, *(iii)* final exams of academic institutions in astronomy, *(iv)* online encyclopedias, and *(v)* manual construction by ourselves. In addition, we confirm that our datasets do not involve any copyright infringement and are free to use and distribute under the CC-BY-4.0 license. Next, we will describe how the questions are obtained and processed in more detail.

#### Existing Benchmarks

Several benchmarks for LLMs in the general domain have contained some questions about astronomy. Astro-QA reuses the questions from the astronomical sections of three public general-domain benchmarks: MMLU^21^, CMMLU^22^, and CG-Eval^24^. These sources provide curated astronomical questions with ground-truth answers and thus can be directly used in Astro-QA. Specifically, we obtain 136 single-select multiple-choice questions from MMLU and 167 single-select multiple-choice questions from CMMLU, respectively. Then, we acquire 80 terminology and 76 short-answer questions from CG-Eval. In particular, during the collection process, we thoroughly reviewed these questions and identified a few errors that were corrected to ensure the credibility of the data. We also reported them to the authors of these benchmarks.

#### Astronomy Olympiads

Several renowned astronomical competitions, including the United States Astronomy and Astrophysics Olympiad (USAAAO, https://usaaao.org) and the Chinese National Astronomy Olympiad (CNAO, https://www.bjp.org.cn/qgzxstwzsjs/list.shtml), publish a collection of questions along with answers on their websites. Specifically, we used a total of 488 (single-select and multi-select) multiple-choice questions from these Olympiads.

#### Final Exams of Universities and Astronomical Institutions

We gathered and organized the questions in the final exams of universities and astronomical institutions, which were publicly available on their websites. After crawling the source PDF files from web pages, we manually reviewed and distilled 1,065 (single-select and multi-select) multiple-choice and 317 judgment questions.

#### Online Encyclopedias

We devised subjective questions by curating pages related to astronomy from two online encyclopedias: Wikipedia (https://www.wikipedia.org/) and Baidu Baike (https://baike.baidu.com/). As a result, we manually constructed 243 terminology and 155 short answer questions, whose ground-truth answers are their corresponding content on the page edited by crowdsourced workers.

#### Manual Construction

Due to the lack of matching questions in astronomy from the above sources, we constructed 355 matching questions manually. Specifically, this was done by taking statements relevant to similar knowledge from multiple-choice questions as a group, splitting them into two to four parts, and then disturbing their order. Our main selection criteria were that the questions were answered succinctly and that the answers were unique. In the data calibration session, our manual work focused on solving problems with ambiguous references, formula writing normality, and the correctness of answers. We chose to discard certain questions, specifically, questions with indeterminable or unclear answers and questions that were repetitive or unrelated to astronomy.

#### License Issues

The licenses for the above data sources are listed as follows:

- For existing benchmarks, CG-Eval and CMMLU follow the CC BY-NC-SA 4.0 license, and MMLU follows the MIT license.
- For USAAAO, CNAO, and university exams, the websites for publication include sharing agreements, which indicates that the questions are not copyrighted and are freely available for public use.
- Wikipedia and Baidu Baike also follow the CC BY-SA 4.0 license.

Therefore, publishing the dataset under the CC-BY-4.0 license is legal without any copyright infringement.

### Data Processing

The construction of the Astro-QA dataset was divided into four main steps: data collection, data calibration and correction in two rounds, and answer explanation addition. The crowdsourced workers include 30 professors and graduate students from our research team, as well as several other teams working in collaboration with us. Their roles and responsibilities are listed below.

- Four professors in the cross-disciplinary field of astronomy and computer science, defined as first-level workers.
- Four graduate students with at least two years of experience in the intersection of the LLMs and the astronomical field and two graduate Ph.D. students in the intersection of the LLMs and the astronomical field, defined as second-level workers.
- The remaining graduate students in the field of computer science, defined as third-level workers.

The data collection process requires the workers to have relevant experience in both the LLM and astronomy fields. Thus, second-level workers screen credible data sources, determine their copyright information, and then perform in-group cross-validation.

In the first round of data validation, we mainly check the content’s correct answers and formula writing standardization. This session is handed over to third-level workers for calibration and is finally reviewed by second-level workers. Problems found during the review are recorded in a timely manner. The second round of data validation and correction focused on correcting the issues that arose in the first round and giving each answer as much resolution as possible. This process is led by a second-level worker as a team leader, who is responsible for solving most of the problems and referring those that cannot be solved to a first-level worker.

After collecting and constructing the questions and their answers, we manually add a comprehensive explanation of the answer to each question. This was done by recruiting crowdsourced annotators with essential knowledge of astronomy. Each worker was presented with a question and its answer at a time and asked to search web pages, review reference books, and discuss with other workers to write an explanation of how the answer was reached. Each explanation was cross-validated by another worker for its quality and reliability.

All first- and second-level workers have at least two years of research experience in astronomy and AI. Although third-level workers may lack experience in the field of astronomy, we provided them with professional training prior to data calibration and developed detailed data collation standards as a guide. These criteria include:

- Scrutinizing each question in the dataset for availability, correctness of answers, and normality of formula writing;
- Retrieving and verifying answers in a way that is limited to searching for the source of the question through books, the Internet, etc., and checking the officially provided answers and explanations;
- The use of LLMs to assist in the completion of the work is strictly prohibited;
- Provide timely feedback to the secondary worker if there are instances of unclear judgment of answers or questions. In terms of compensation, we pay each participant in the form of a labor fee according to their total work hours. The mentor teams volunteered to participate without pay.

To reduce the likelihood of data being unintentionally incorporated into the pre-training corpora of models, we avoided using the original text from Wikipedia and Baidu Baike pages or other publicly available online content as answers for questions related to astronomical terminology and other subjective questions. Instead, we invited experts in the field of astronomy to restate the content of the original articles.

**Fig. 2:**
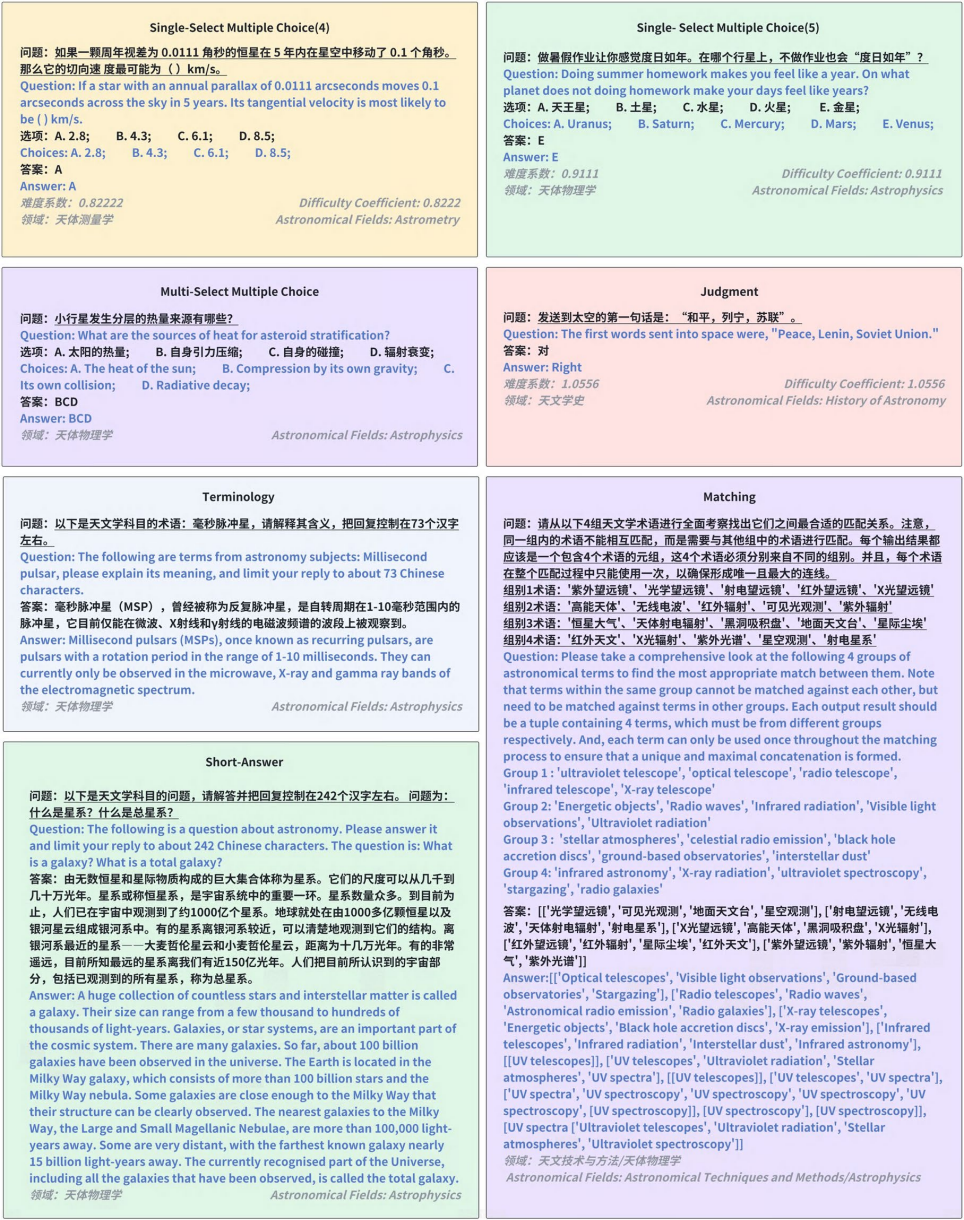
Illustrative examples of different question types in the Astro-QA dataset.

We present an example for each type of question in the Astro-QA dataset in Fig. 2.

### Prompt Design

**Fig. 3:**
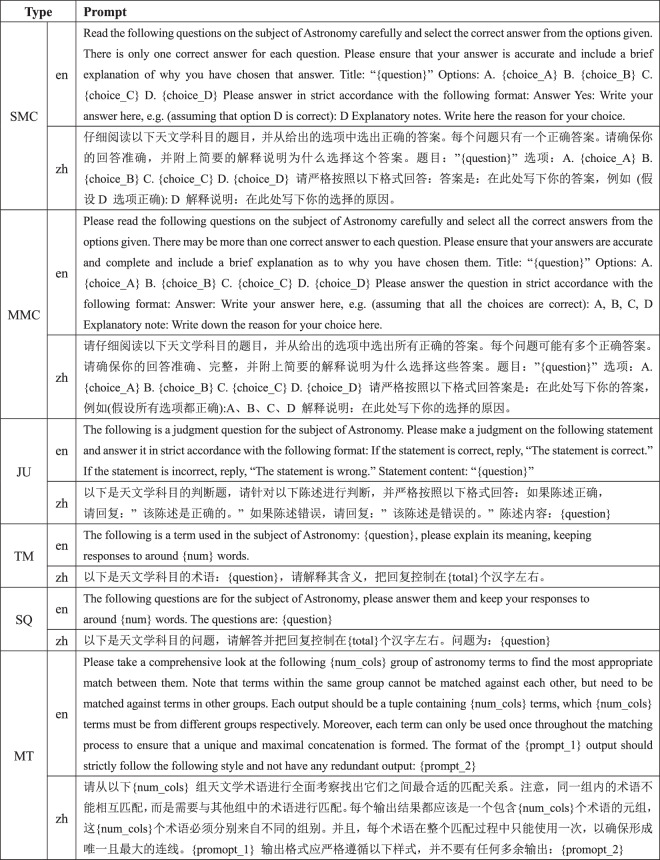
Prompt templates in English and Chinese for each question type.

**Fig. 4:**
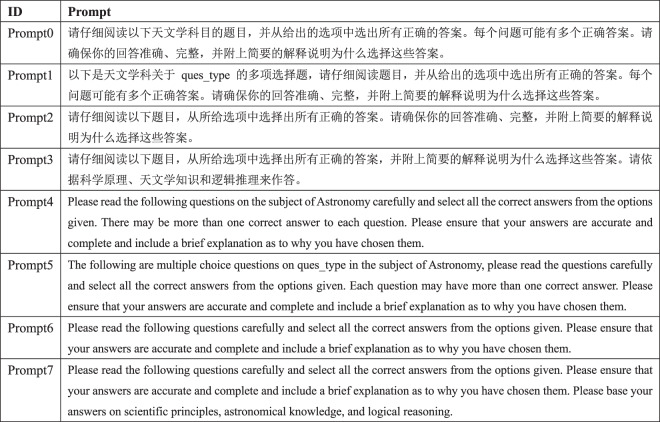
Prompt templates for multiple choice questions used in the prompt sensitivity experiments.

LLMs operate in a prompt-driven mode, where responses are generated based on input prompts provided by users. As such, we need to design prompts for LLMs to understand and respond appropriately to questions. For fair comparison, we design a uniform and standardized prompt template for each type of question, which are fed to all LLMs. The details of these prompt templates in English and Chinese are outlined in Fig. 3. Due to the fact that model responses may be sensitive to the choice of prompts, we design eight additional prompt templates, four in Chinese and four in English, specifically for multiple choice questions in Fig. 4 to test the sensitivity of LLMs.

## Data Records

**Fig. 5:**
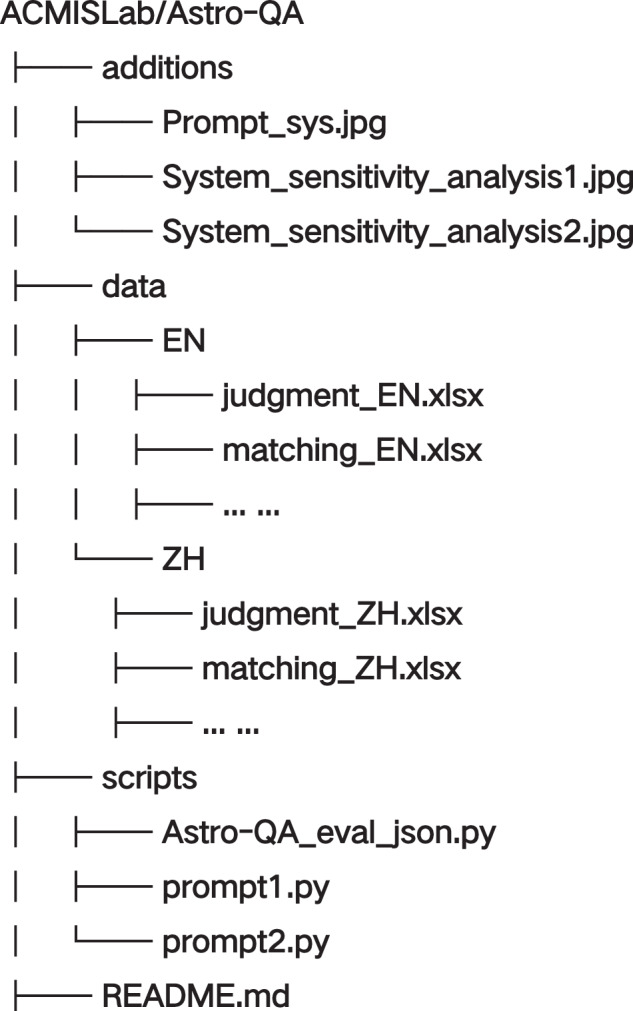
Illustration of the structure of the GitHub repository directory.

The Astro-QA dataset is available on both GitHub (https://github.com/ACMISLab/Astro-QA) and Figshare^25^ and is distributed under the CC BY 4.0 license. The structure of the repository directory is shown in Fig. 5. The folder ‘data’ contains all dataset files for evaluation, in which the two sub-folders, ‘EN’ and ‘ZH’, include the English and Chinese versions of the questions, respectively. Then, the questions are organized by types in .xlsx format. For each question file, there are several key headers: ‘ID’ is the unique identifier of each question; ‘question’ denotes the body of the evaluation question; ‘answer’ provides the standard (reference) answer to the question; and ‘parse’ offers an explanation of the question. The folder ‘scripts’ contains the scripts for evaluation, which can be run directly for LLM evaluation and calculate the overall performance score based on the answers. The folder ‘additions’ contains the prompt template used in our experiments, as well as the files related to the comparison experiments on system prompt sensitivity analysis, including both prompts and experimental results. Specifically, the file ‘Prompt_sys.jpg’ is the prompt word settings in English and Chinese; the files ‘System_sensitivity_analysis1.jpg’ and ‘System_sensitivity_analysis2.jpg’ are the experiment results for the sensitivity of system prompts. For more detailed information, please refer to the file ‘README.md’ which provides a comprehensive description of the repository.

## Technical Validation

### Experimental Setup

#### Environment

The experiments with open-source models were conducted on a server with eight NVIDIA RTX A6000 GPUs. For commercial models, we used their official APIs for evaluation in the experiments. We adopted the zero-shot prompt setting in all experiments.

#### Tested LLMs

We evaluated 27 pre-trained LLMs that span a range of parameter sizes from 6B to 236B, including both open-source and commercial models, on the Astro-QA dataset. Especially, the following LLMs are tested: (1) GPT series^1^, including GPT-3.5 Turbo, GPT-4 Turbo, GPT-4o; (2) GLM series^3^, including ChatGLM2-6B, ChatGLM3-6B, and commercial GLM-4; (3) Baichuan series^26^, including Baichuan2-7B and Baichuan2-13B; (4) InternLM series^27^, including InternLM2-7B and InternLM2-20B; (5) Qwen1.5 series^28^ ranging from 7B to 110B parameters and commercial Qwen-MAX; (6) StarWhisper3^12^ based on Qwen-14B fine-tuned for the astronomical domain; (7) Llama3 series^2^, including Llama3-8B and Llama3-70B; (8) AstroMLab series^10,11^, including AstroSage-8B and AstroLLaMA-2-70B, based on Llama-3.1-8B and LLaMA-2-70B and fine-tuned in the astronomical domain; (9) Google GeMini-Pro^29^; and (10) SparkV3.5 (https://xinghuo.xfyun.cn/spark). Sparked by Mixtral^30^, mixture-of-expert (MoE) models have recently gained significant attention. Therefore, we tested the following MoE models: (11) Qwen1.5-MoE-A2.7B^31^; (12) commercial Mixtral 8 × 7B and Mixtral 8 × 22B; and (13) DeepSeek-V2^32^.

A comprehensive overview of the models evaluated is presented in Table 2. The column “#Params” indicates their respective parameters. The column “#Tokens” reveals the amount of pre-training data used by the model. The column “Window Size” reveals the size of the model context window. The column “Open” indicates whether the model is open-source or commercial. The column “Access” indicates how we test the model, either through web interfaces and APIs or through their weights. Finally, a dashed line (‘-’) represents an unknown value.

**Table 2:** Detailed information of the models evaluated in the experiments.

Model	#Params	#Tokens	Window Size	Open	Access
ChatGLM2-6B	6.2B	1.4T	8K	*✓*	API
ChatGLM3-6B	6.2B	—	8K	*✓*	API
Baichuan2-7B	7B	2.6T	4K	*✓*	API
Qwen1.5-7B	7B	—	32K	*✓*	API
InternLM2-7B	7B	—	200K	*✓*	Weights
Llama3-8B	8B	> 15T	8K	*✓*	Weights
AstroSage-8B	8B	3.3B	8K	*✓*	Weights
Baichuan2-13B	13B	2.6T	4K	*✓*	API
Qwen1.5-14B	14B	—	32K	*✓*	API
StarWhisper3	14B	—	32K	*✓*	Weights
InternLM2-20B	20B	—	200K	*✓*	Weights
Qwen1.5-MoE-A2.7B	14B	—	32K	*✓*	Weights
Qwen1.5-32B	32B	—	32K	*✓*	API
Mixtral 8 × 7B	47B	—	32K	*✓*	API
Llama3-70B	70B	> 15T	8K	*✓*	API
AstroLLaMA2-70B	70B	—	4K	*✓*	Weights
Qwen1.5-72B	72B	—	32K	*✓*	API
Qwen1.5-110B	110B	—	32K	*✓*	API
Mixtral 8 × 22B	141B	—	32K	*✓*	API
DeepSeek-V2	236B	8.1T	32K	*✓*	API
GeMini-Pro	—	—	32K	✕	API
SparkV3.5	—	—	8K	✕	API
GLM-4	—	—	128K	✕	API
Qwen-MAX	—	—	8K	✕	API
GPT-3.5 Turbo	—	—	16K	✕	API
GPT-4 Turbo	—	—	128K	✕	API
GPT-4o	—	—	128K	✕	API

#### Parameter and Prompt Settings

The parameters for reasoning with LLMs are set as follows: top_k=50, top_p=0.8, temperature=0.7 to 0.85, and repetition_penalty=1. Among these parameters, the results are most sensitive to the temperature parameter. As the temperature value approaches 0, the model responses tend to be more stable; conversely, they are more open. In the experiments, we aim to test the generalizability of the LLM responses. Therefore, we set the temperature in an interval that will show a better generalization. In this way, we reduce the effect of errors that occur in a single experiment on the overall performance of an LLM.

For each task, we apply a standardized zero-shot prompt template. As shown in Fig. 3, we instructed the LLMs that this was the topic of the astronomy subject and tailored different prompts for each task adapting to its specific inputs and outputs. The LLM responses should contain an explanation of the response. Taking into account the variations of the LLM responses, we asked each question five times and took the best answer for evaluation.

### Evaluation Measure

We introduce a new measure, *DGscore*, for evaluation. Generally, DGscore integrates a wide spectrum of measures for the six types of question and incorporates a weighting scheme based on type- and question-specific difficulty coefficients to accurately assess the overall performance of an LLM. Next, we describe each part of the DGscore in more detail.

For each objective question, the quality measure of an answer is based solely on its *correctness*. The only exception is for a multi-select multiple-choice (MMC) question, an answer is considered *partially correct* if it contains a part of the correct options but does not contain any incorrect option. In existing benchmarks, the performance of an LLM on objective questions is typically evaluated by *accuracy*, that is, the ratio between the number of correctly answered questions and the number of all questions. However, the accuracy measure ignores the difficulty of different questions and does not apply to MMC questions. To address these issues, we adopt a weighting scheme based on type- and question-specific difficulty coefficients to evaluate the performance of an LLM on the three types of objective questions. The scoring criteria are presented in Table 3. In particular, we first assign a base score to each type of question that reflects its overall difficulty: 0.5 for JU, 1 for SMC(4), 1.2 for SMC(4), 2 for MMC, and 3 for MT. Then, we further introduce a question-specific difficulty coefficient for each JU and SMC question. This coefficient further reflects its difficulty and is calculated as follows:

1 $${{\mathcal{D}}}_{i}=1-\frac{\#{{\rm{TRUE}}}_{i}}{\#{\rm{Models}}}+\varepsilon ,$$

where *i* is the index of a specific question, *#*TRUE_*i*_ is the number of models tested that can correctly answer question *i*, *#*Models is the total number of models tested, and $\varepsilon \in {{\mathbb{R}}}^{+}$ is a factor (typically set to 0.2) to adjust the difficulty coefficient to the range [*ε*, 1 + *ε*]. A coefficient ${{\mathcal{D}}}_{i}$ closer to *ε* indicates a lower difficulty level of question *i*; on the contrary, a coefficient closer to 1 + *ε* signifies a higher level of difficulty of question *i*. Finally, the overall score of an LLM for all SMC and JU questions is calculated as follows:

2 $${{\rm{Score}}}_{\alpha }=\mathop{\sum }\limits_{i=1}^{n}{{\mathcal{D}}}_{i}{b}_{i}{{\mathbb{I}}}_{i},$$

where *n* is the total number of SMC and JU questions, *b*_*i*_ represents the base score according to the type of question *i*, and ${{\mathbb{I}}}_{i}$ indicates whether question *i* is correctly answered by the LLM (${{\mathbb{I}}}_{i}=1$ for correct answer and ${{\mathbb{I}}}_{i}=0$ for incorrect answer). We categorize the SMC(4), SMC(5), and JU questions into three difficulty levels, i.e., *easy* (${{\mathcal{D}}}_{i}\,\in$ [0.2, 0.5]), *normal* (${{\mathcal{D}}}_{i}\,\in$ (0.5, 1]), and *hard* (${{\mathcal{D}}}_{i}\,\in$ (1, 1.2]), based on their difficulty coefficients. In total, the easy questions account for 45.3%, the normal questions account for 42.1%, and the hard questions account for 12.6%. Moreover, ${{\rm{Score}}}_{\beta }$ represents the score of an LLM for the MMC and MT questions. The calculation of ${{\rm{Score}}}_{\beta }$ is similar to that of ${{\rm{Score}}}_{\alpha }$. The only difference is that the coefficient ${{\mathcal{D}}}_{i}$ of each question is omitted in ${{\rm{Score}}}_{\beta }$.

**Table 3:** Scoring criteria of each type of question in Astro-QA.

Type	Scoring Criteria
JU	0.5 multiplied by ${{\mathcal{D}}}_{i}$ for a correct answer; 0 for an incorrect answer
SMC(4)	1 multiplied by ${{\mathcal{D}}}_{i}$ for a correct answer; 0 for an incorrect answer
SMC(5)	1.2 multiplied by ${{\mathcal{D}}}_{i}$ for a correct answer; 0 for an incorrect answer
MMC	2 or 0.5 for a fully or partially correct answer; 0 if containing any incorrect option
MT	3 for a correct answer; 0 if containing any incorrect match
TM	Answer similarity score multiplied by 5
SQ	Answer similarity score multiplied by 5

For subjective questions, existing benchmarks typically adopt three measures of semantic similarity between an answer and the ground truth, namely Bleu, ROUGE, and chrF. Specifically, Bleu^33^, initially designed for machine translation evaluation, underscores the *n*-gram matching but may overlook nuanced semantic differences. ROUGE^34^, proposed for extractive summary evaluation, aims to strike a balance between accuracy and recall but may neglect redundancy and semantic depth. And chrF^35^ provides granular insights at the character level but may overly prioritize surface-level text forms. However, when used individually, these metrics have inherent limitations in assessing text generation quality in a comprehensive way. Gscore^24^ considers addressing the limitations of the above metrics by seamlessly integrating them into a unified measure. Especially, Gscore leverages the matching precision measure in Bleu, the balanced recall and precision measure in ROUGE, and the granular analysis measure in chrF, and introduces an additional similarity measure to capture the semantic relevance of two pieces of text. This multifaceted measure offers a more accurate evaluation of (1) how close a generated answer by an LLM is to the ground truth and (2) how fluent and coherent a generated answer itself is. We adopt the same measure as the Gscore to evaluate the quality of answers to objective questions. The final score for subjective questions in Astro-QA is presented as follows:

3 $$\begin{array}{l}{{\rm{Score}}}_{\gamma }=(0.3\times {{\rm{B}}{\rm{L}}{\rm{E}}{\rm{U}}}_{\gamma }+0.1\times {{\rm{ROUGE1}}}_{\gamma }+0.15\times {{\rm{ROUGE2}}}_{\gamma }\\ \,\,\,+\,0.1\times {{\rm{ROUGEL}}}_{\gamma }+0.35\times {{\rm{C}}{\rm{H}}{\rm{R}}{\rm{F}}}_{\gamma })\times b\end{array}$$

where *b* represents the base score for subjective questions and is set to 5 in the DGscore. Note that the coefficients in Eq. (3) are determined empirically and can be changed to other positive values whose sum equals 1.

Finally, the DGscore of an LLM is calculated as the sum of the scores for different types of questions:

4 $${\rm{DGscore}}={{\rm{Score}}}_{\alpha }+{{\rm{Score}}}_{\beta }+{{\rm{Score}}}_{\gamma }.$$

### Experimental Results

#### Main Results

**Fig. 6:**
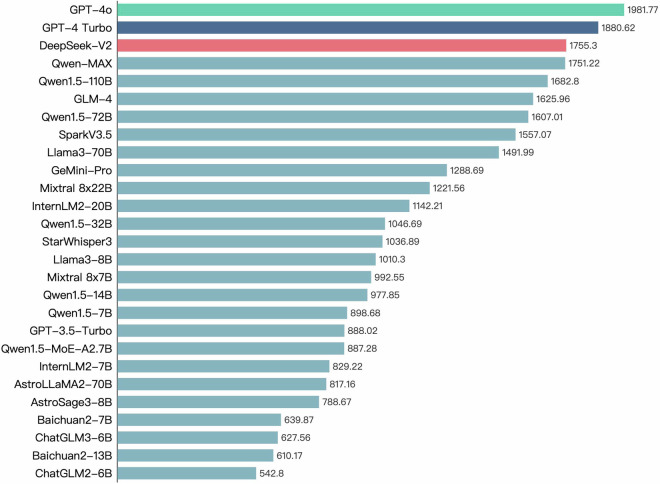
Ranking of all the LLMs tested on the Astro-QA dataset based on the DGscore.

Fig. 6 shows the ranking of all LLMs tested on the Astro-QA dataset based on their DGscores. We observe that GPT-4o emerges as the top performer, highlighting its exceptional comprehensive and generative ability in astronomy. Among open-source models, Qwen1.5-72B and Qwen1.5-110B perform remarkably well in the astronomy domain, even surpassing some commercial models. Furthermore, fine-tuned in the astronomy domain, StarWhisper3 exhibits significantly improved performance compared to its base model, Qwen1.5-14B. This indicates that using specialized astronomical data for model fine-tuning can lead to significant gains in QA performance. Meanwhile, the MoE models represented by DeepSeek-V2 also demonstrated their distinctive capacity. By integrating knowledge from various professional areas, the MoE models present a novel and efficient LLM solution, thereby expanding their application in scientific domains. These results indicate that the Astro-QA dataset is comprehensive and that the ranking based on the DGscore can accurately reflect the QA capacity of LLMs in astronomy.

#### Results for Objective Questions

Table 4 presents the results of all LLMs for each type of objective question. Specifically, for the SMC(4), SMC(5), JU, and MT questions, we use the accuracy ratios of the answers for evaluation. For MMC questions, the evaluation is based on the total scores according to the criterion in Table 3. GPT-4o has exhibited exceptional performance across all types of questions, particularly in MMC questions, where it achieves an impressive score of 107.4 that significantly outperforms other LLMs. Furthermore, GPT-4 Turbo and GPT-4o achieve accuracy rates greater than 90% in the MT questions. For the SMC(4) and SMC(5) questions, GPT-4o also stands out with accuracy rates of 0.86. These results demonstrate that GPT-4 series show outstanding performance on complex and challenging questions, demonstrating their powerful capabilities in comprehensive processing and multi-dimensional reasoning. We note that specialized astronomical LLMs, particularly the AstroLLaMA series, do not perform well on Astro-QA for all types of questions except JU, even falling short of general-purpose LLMs with the same parameter scale. This highlights that there is still significant room for improvement in the development of astronomical LLMs. Other LLMs, such as the GLM and Baichuan series, only achieve about 10% accuracy rates on MT questions, which indicates that despite their ability to understand language tasks, they still exhibit a significant deficiency in accurately distinguishing and matching concepts in complex contexts and with high semantic correlations. Generally, we observe that the performance of LLMs on the Astro-QA dataset improves with the number of parameters, following the scaling law^36^.

**Table 4:** Evaluation results of all the LLMs on the Astro-QA dataset for objective questions.

#Params	Model	SMC(4)	SMC(5)	MMC	JU	MT
6B–10B	ChatGLM2-6B	0.30	0.38	34.0	0.51	0.07
	ChatGLM3-6B	0.41	0.38	49.0	0.64	0.06
	Baichuan2-7B	0.36	0.27	41.5	0.51	0.13
	Qwen1.5-7B	0.57	0.61	59.9	0.66	0.30
	InternLM2-7B	0.66	0.68	49.2	0.62	0.50
	Llama3-8B	0.57	0.56	57.8	0.58	0.60
	AstroSage-8B	0.56	0.52	63.4	0.88	0.30
10B–30B	Baichuan2-13B	0.47	0.41	32.7	0.58	0.10
	Qwen1.5-14B	0.61	0.65	74.8	0.69	0.37
	StarWhisper3 (14B)	0.62	0.67	77.0	0.69	0.41
	InternLM2-20B	0.75	0.76	71.9	0.70	0.67
	Qwen1.5-MoE-A2.7B	0.60	0.62	55.6	0.74	0.23
30B–100B	Qwen1.5-32B	0.67	0.73	93.8	0.74	0.37
	Mixtral 8 × 7B (47B)	0.48	0.42	64.8	0.66	0.55
	Llama3-70B	0.73	0.75	89.4	0.82	0.81
	AstroLLaMA2-70B	0.54	0.61	48	**0.97**	0.3
	Qwen1.5-72B	0.74	0.78	94.5	0.83	0.76
100^+^B	Qwen-110B	0.75	0.81	104.1	0.84	0.73
	Mixtral 8 × 22B (141B)	0.60	0.59	70.4	0.62	0.72
	DeepSeek-V2 (236B)	0.75	0.78	85.2	0.82	0.78
n/a	GeMini-Pro	0.76	0.74	76.5	0.88	0.77
	SparkV3.5	0.80	0.80	82.5	0.80	0.73
	GLM-4	0.71	0.72	87.9	0.76	0.85
	Qwen-MAX	0.73	0.81	87.1	0.81	0.80
	GPT-3.5 Turbo	0.68	0.68	69.0	0.84	0.45
	GPT-4 Turbo	0.77	0.80	88.6	0.79	0.91
	GPT-4o	**0.86**	**0.86**	**107.4**	0.88	**0.94**

The bolded text indicates the highest score for each question type among all LLMs and the underlined text indicates the highest score for each question type in each group of LLMs whose parameter numbers are in the same scale.

#### Results for Subjective Questions

Table 5 presents the results of all LLMs for two types of subjective questions. Specifically, we list the detailed scores of Bleu, ROUGE-1, ROUGE-2, ROUGE-L, and chrF, which are integrated in the calculation of the DGscore. For TM questions, different from the results for objective questions, we observe that SparkV3.5 achieves the best performance among all LLMs. It can generate more professional explanations of terms relevant to astronomy. We think that the reason for its superior performance is that its training corpus contains more scientific documents in Chinese. The high Bleu score for the TM questions signifies that the responses provided by SparkV3.5 match the key phrases and terms in the reference answers. SparkV3.5 also excels in the ROUGE and chrF scores, indicating that it maintains semantic fluency and coherence in the generated text. For SQ questions, several LLMs, including SparkV3.5, Qwen-MAX, GPT-4 Turbo, and GPT-4o, show close performance to each other and Qwen-MAX is top-ranked with a marginal advantage. Finally, we observe that all LLMs show inferior performance on subjective questions: the generated text often contains factual errors and lacks coherence and vocabulary richness. This means that there is still a lot of room for improvement in applying LLMs to open-ended astronomical QA.

**Table 5:** Evaluation results of all the LLMs on the Astro-QA dataset for subjective questions.

#Params	Model	TM					SQ				
		Bleu	R-1	R-2	R-L	chrF	Bleu	R-1	R-2	R-L	chrF
6B–10B	ChatGLM2-6B	22.05	27.04	6.01	20.90	9.71	22.38	29.07	8.91	23.01	13.03
	ChatGLM3-6B	25.08	26.90	6.28	19.49	11.36	24.84	29.40	8.95	22.64	13.61
	Baichuan2-7B	25.37	28.89	7.11	21.60	11.12	24.80	30.79	9.51	23.87	13.80
	Qwen1.5-7B	29.11	29.29	6.35	20.50	11.15	28.35	32.20	9.00	23.70	13.27
	InternLM2-7B	30.18	29.52	7.18	21.02	12.08	30.66	33.55	10.96	26.12	16.27
	Llama3-8B	29.79	29.32	6.86	21.48	13.55	30.04	30.09	8.58	22.96	13.80
	AstroSage-8B	20.91	25.09	2.47	19.74	8.25	19.37	24.87	2.66	20.08	7.44
10B–30B	Baichuan2-13B	12.41	26.07	4.72	20.48	7.19	19.07	29.00	7.10	22.47	10.25
	Qwen1.5-14B	28.43	31.10	7.22	22.46	11.06	28.64	33.73	9.80	24.80	13.68
	StarWhisper3 (14B)	26.29	27.76	6.14	18.34	12.87	29.15	30.66	9.36	21.23	16.16
	InternLM2-20B	29.52	29.66	7.17	20.78	12.99	30.82	33.45	10.76	25.62	16.58
	Qwen1.5-MoE-A2.7B	29.21	28.56	7.05	19.37	12.77	28.09	31.75	10.18	22.85	17.01
30B–100B	Qwen1.5-32B	25.50	31.35	7.47	23.14	10.70	27.67	34.08	10.78	25.78	14.53
	Mixtral 8 × 7B (47B)	19.90	23.19	5.14	16.19	11.65	25.32	26.51	7.30	19.51	12.80
	Llama3-70B	28.20	28.61	7.01	21.53	12.93	28.81	31.49	10.08	24.48	14.03
	AstroLLaMA2-70B	15.84	25.60	2.33	21.65	8.04	19.02	27.61	3.73	23.03	11.30
	Qwen1.5-72B	24.88	31.94	8.06	24.24	10.71	28.18	34.82	11.03	26.46	14.43
100^+^B	Qwen1.5-110B	24.46	32.40	8.30	24.09	10.86	25.52	34.23	10.36	25.71	13.31
	Mixtral 8 × 22B (141B)	29.73	28.67	6.66	20.21	12.47	30.95	31.67	9.39	23.64	14.74
	DeepSeek-V2 (236B)	32.29	31.22	8.34	21.81	13.65	33.56	34.13	11.07	25.22	16.14
n/a	GeMini-Pro	30.19	29.95	7.28	20.67	12.92	29.38	31.33	8.95	23.09	13.98
	SparkV3.5	**33.70**	32.53	**10.03**	23.64	**14.48**	30.40	34.12	**12.24**	25.47	**18.27**
	GLM-4	20.86	**33.60**	9.47	**26.10**	11.01	23.78	34.86	11.77	27.03	14.62
	Qwen-MAX	31.78	32.09	8.40	23.14	12.55	32.97	35.14	11.85	26.23	16.73
	GPT-3.5 Turbo	29.00	27.66	6.84	19.50	11.65	27.17	31.30	9.43	22.77	14.07
	GPT-4 Turbo	33.25	31.68	7.96	22.55	12.62	**34.11**	34.43	11.48	25.79	15.79
	GPT-4o	27.89	32.55	8.12	24.08	11.49	28.46	**35.22**	11.14	**27.21**	14.30

The bolded text indicates the highest score for each question type among all LLMs and the underlined text indicates the highest score for each question type in each group of LLMs whose parameter numbers are in the same scale. Note that ‘R’ is short for ‘ROUGE’.

#### Impact of Prompts on LLMs

To evaluate the impact of prompts on LLM responses, we designed eight different prompts in Chinese and English in Fig. 4 to perform sensitivity analysis experiments. We also performed experiments with and without system prompts. The system prompt “You are an expert in astronomy.” was used in the experiments. As commercial LLMs are continuously updated, we tested the five LLMs that were deployed locally, namely InternLM-7B, InternLM-20B, Llama3-8B, Qwen1.5-MoE-A2.7B, and StarWhisper3, in the experiments to obtain consistent results. The experimental results are presented in Table 6. When system prompts are not used, InternLM-20B, Llama3-8B, and StarWhisper3 exhibited minor score fluctuations but no significant improvements. This indicates that these models demonstrate relatively stable performance with low sensitivity to prompts when system prompts are not provided. In addition, InternLM-7B and Qwen1.5-MoE-A2.7B show noticeable improvements in the scores on English prompts compared to Chinese ones. When system prompts are used, the scores of the five models fluctuated only slightly, indicating overall stability. In particular, Llama3-8B, Qwen1.5-MoE-A2.7B, and StarWhisper3 showed improved performance with system prompts, suggesting that system prompts guide models to generate higher quality responses. InternLM-7B and InternLM-20B experienced significant score drops when system prompts were used. The underlying reasons may involve how the models parse system prompts or the compatibility between prompts and the models’ internal knowledge bases.

**Table 6:** Experimental results for LLMs for different prompts (with and without system prompts). ‘P*i*’ means that Prompt*i* in Fig. 4 is used for evaluation.

System Prompt	Model	P0	P1	P2	P3	P4	P5	P6	P7	Std. Dev.
Default	InternLM-7B	49.2	43.6	48.1	52.1	58	53.8	**61.7**	59.6	6.23
	InternLM-20B	71.9	70.3	69.6	69	70.9	**74.4**	65	69.6	2.68
	Llama3-8B	57.8	58.8	52.4	51.6	57	**61.4**	52.2	55.5	3.55
	Qwen1.5-MoE-A2.7B	55.6	57.8	48.7	64	66.9	63.3	**67.5**	66.2	6.64
	StarWhisper3	77	**80.3**	76.2	78	75.1	79.1	75	73.6	2.26
"You are an expert in astronomy.”	InternLM-7B	34	34.7	32.9	33.6	33.7	33	33.5	**35.7**	0.92
	InternLM-20B	33.2	31.9	31.5	**33.4**	30	33	31.4	29.8	1.39
	Llama3-8B	58.8	**59.8**	56.7	53	58.7	58	57.7	54.9	2.26
	Qwen1.5-MoE-A2.7B	57.6	63.9	55.4	64.3	63.8	60.8	68.3	**69.6**	4.88
	StarWhisper3	78.9	**79.6**	77	78	74.3	78.8	74.6	75.6	2.05

## Usage Notes

The primary objective of this study is to present the Astro-QA dataset to benchmark the question-answering (QA) capacity of LLMs in the astronomical domain. To facilitate researchers to use our dataset, we develop several scripts in Python to perform the benchmarks and include them in the folder ‘scripts’ of the GitHub repository. As shown in Fig. 5, ‘prompt1.py’ is the evaluation script for the *single-select multiple-choice (4)*, *single-select multiple-choice (5)*, *multi-select multiple-choice*, *judgment*, *terminology*, and *short-answer* questions. Then, ‘prompt2.py’ is the evaluation script for the *matching* questions. After collecting the results, the DGscores can be automatically computed by running the script ‘Astro - QA_eval_json.py’. For more detailed guidelines and usage examples, please refer to the file ‘README.md’ of the repository.

## Data Availability

The entire process, including the construction of the dataset and the conduction of experiments, was implemented using the Python programming language. The dataset is hosted on Figshare^25^ and the code is hosted on GitHub (https://github.com/ACMISLab/Astro-QA).
